# Changes in Mitochondrial Genome Associated with Predisposition to Atherosclerosis and Related Disease

**DOI:** 10.3390/biom9080377

**Published:** 2019-08-18

**Authors:** Aleksandrina Volobueva, Andrey Grechko, Shaw-Fang Yet, Igor Sobenin, Alexander Orekhov

**Affiliations:** 1 Laboratory of Gene Therapy, Biocad Biotechnology Company, Saint-Petersburg, Strelnya 198515, Russia; 2 Federal Scientific Clinical Center for Resuscitation and Rehabilitation, Moscow 109240, Russia; 3 Institute of Cellular and System Medicine, National Health Research Institutes, 35 Keyan Road, Zhunan Town, Miaoli County 35053, Taiwan; 4 Laboratory of Medical Genetics, Russian Cardiology Research and Production Complex, Moscow, Russia; 5 Institute for Atherosclerosis Research, Skolkovo Innovative Center, Moscow 121609, Russia; 6 Institute of Human Morphology, Moscow 117418, Russia; 7 Institute of General Pathology and Pathophysiology, Moscow 125315, Russia

**Keywords:** atherosclerosis, mitochondria, mtDNA, mutations, heteroplasmy, molecular markers

## Abstract

Atherosclerosis-related cardiovascular diseases remain the leading cause of morbidity and mortality, and the search for novel diagnostic and therapeutic methods is ongoing. Mitochondrial DNA (mtDNA) mutations associated with atherosclerosis represent one of the less explored aspects of the disease pathogenesis that may bring some interesting opportunities for establishing novel molecular markers and, possibly, new points of therapeutic intervention. Recent studies have identified a number of mtDNA mutations, for which the heteroplasmy level was positively or negatively associated with atherosclerosis, including the disease at its early, subclinical stages. In this review, we summarize the results of these studies, providing a list of human mtDNA mutations potentially involved in atherosclerosis. The molecular mechanisms underlying such involvement remain to be elucidated, although it is likely that some of them may be responsible for the increased oxidative stress, which plays an important role in atherosclerosis.

## 1. Introduction

Atherosclerotic lesions can develop in virtually any artery and are associated with some of the most prevalent cardiovascular disorders (CVDs) that are the leading causes of morbidity and mortality. Atherosclerosis-associated CVDs include coronary heart disease (CHD) (coronary artery disease, CAD), coronary microvascular disease, carotid artery disease, peripheral artery disease, and chronic kidney disease. Despite the common belief, atherosclerosis occurrence is not limited to the elderly population. The disease is common in young people and, in many cases, has no clinical signs at early stages. Atherosclerosis can remain asymptomatic for decades before causing such serious events as a myocardial infraction or stroke. This makes atherosclerosis a challenging problem of modern medicine, with an urgent need for novel relevant biomarkers to facilitate early diagnostics.

The mechanism of atherosclerosis development is quite complex and includes several processes: Low-density lipoprotein (LDL) oxidation, inflammation, cellular adhesion, fibrosis, and arterial wall calcification [1]. Atherosclerotic plaques are frequently formed at the sites of endothelial damage and accumulation of circulating lipids, such as bends or bifurcations of the blood vessels. Modified low-density lipoprotein (LDL) appears to be the main source of lipid accumulation in the plaques. After being chemically modified, for instance, oxidized by reactive oxygen species (ROS), LDL can become immunogenic and induce the formation of immune complexes. Moreover, self-associates of modified LDL particles can reach a relatively large size. Presence of these large associates in the blood results in their accumulation in the arterial wall cells, since they cannot be processed by regular ways dependent on receptor-mediated endocytosis [2]. The accumulating data positioned atherosclerosis as a chronic inflammatory condition and autoimmune disease [3]. Modified LDL can also participate in monocyte activation and recruitment to the lesion site [4]. Macrophages differentiated from monocytes release pro-inflammatory factors and stimulate migration and proliferation of vascular endothelial muscle cells, which leads to enhanced extracellular matrix secretion. Accumulating extracellular matrix material forms the fibrous cap of the atherosclerotic plaque. This cap can be considered to have a protective role, since its rupture leads to thrombus formation, which can have fatal consequences if the thrombus blocks some vital blood vessel [5].

Mitochondria are key cellular organelles responsible for energy production via cellular ATP generation by oxidative phosphorylation. Impaired mitochondrial function is observed in many human pathologies ranging from neurological disorders to CVD and metabolic syndrome [6]. Mitochondria are semi-autonomous organelles of presumably bacterial origin that possess their own genome in the form of small circular DNA (mtDNA). The improvement of molecular biology tools made it possible to study mtDNA genetic variants and mutations and their association with human diseases. To date, numerous such associations have been found, especially connected to neurodegenerative disorders [7]. Mitochondria are now being considered as important players in the disease phenotype formation and possible points of therapeutic intervention. Somatic mtDNA mutations were also found to be associated with various human cancers [8]

Association between mtDNA mutations or damage and key features of atherosclerosis, such as inflammation, apoptosis, and oxidative stress, has been demonstrated [9]. The free radical theory of ageing considers reactive oxygen species (ROS) production as one of the causes of mtDNA damage and respiratory chain impairment, which further increases ROS formation and leads to oxidative stress. This can lead to increased LDL oxidation and therefore formation of atherogenic LDL species. Moreover, mitochondria are actively involved in lipid metabolism. Mitochondrial dysfunction promotes lipolysis and the increase of serum free fatty acids and glycerol [10]. This makes mitochondria important players in atherosclerosis initiation and development.

Each mitochondrion contains dozens of mtDNA copies that are transferred via mitochondrial division and are therefore inherited via maternal line. mtDNA is a circular molecule, which contains 37 genes and several non-coding sequences. Compared to nuclear DNA, mtDNA has a higher mutation rate probably due to higher exposure to ROS, lack of chromatin, and significantly less efficient repair mechanisms. There are two types of mtDNA mutations: homoplasmic, when an organelle contains identical DNA copies, and heteroplasmic, when a mixture of mutated and wild type mtDNA molecules is present in the same organelle. There is also a threshold for mutational load for inducing mitochondrial dysfunction, which varies between mutations. The level of heteroplasmy can be measured using a number of approaches that are explained in detail elsewhere [11].

During the recent years, our group actively studied mtDNA mutations associated with atherosclerosis both in the arterial wall cells and in circulating lymphocytes. In this article, we provide an overview of the available studies on this topic and bring together the main outcomes of our own research. We created a list of relevant mtDNA mutations revealed so far and provide the evidence of interrelation between mtDNA changes and the development of atherosclerotic lesions.

## 2. Changes of mtDNA Detected in Leukocytes and Arterial Wall Cells

A number of studies have been performed on human material, such as white blood cells and arterial wall tissue autopsy samples, obtained from patients with atherosclerosis-associated diseases. Both arterial wall cells of the subendothelial intimal layer and blood cells, especially monocytes-macrophages, participate in atherosclerotic plaque formation. Increased mtDNA heteroplasmy of certain mutations may lead to cell dysfunction due to local increase of oxidative stress promoting atherosclerotic lesion formation.

### 2.1. mtDNA Mutations Discovered in Studies Using Human Arterial Wall Samples

Studies of mtDNA mutations in the arterial wall cells are limited by the need of obtaining post-mortem material but have the advantage of collecting the samples directly from morphologically characterized atherosclerotic plaques. An early study conducted in Italy identified a “common deletion” mtDNA4977 in smooth muscle cells samples from atherosclerotic lesions in abdominal aorta [12]. Its frequency increased with patients’ age. In a more recent study, heteroplasmy level in the homogenates of thoracic aorta intima tissue, from autopsy samples collected after sudden death, was assessed using RT-PCR and pyrosequencing method [13]. Analysis of 40 previously identified mutations in samples from atherosclerotic lesions and unaffected sites of the arterial wall led authors to the conclusion that at least five single nucleotide changes were associated with atherosclerotic lesions. These mutations were A1555G, C3256T, T3336C, G13513A, and G15059A. Furthermore, the study of heteroplasmy level of previously known mutations A1555G, C3256T, G12315A, and G15059A in thoracic aorta samples of 12 male patients demonstrated their higher prevalence in atherosclerotic plaques [14]. These findings possibly support the monoclonal hypothesis of atherosclerosis, according to which somatic mutation occurs in a single muscle cell that further proliferates and produces clonal mutated cells that form the atherosclerotic plaque.

A comparative study of heteroplasmy level in unaffected tissue and atherosclerotic lesions of varying severity was performed on 265 segments of morphologically mapped aortic walls obtained from five individuals [15]. The studied areas were classified as normal tissue, fatty infiltration, fatty streak, lipofibrous plaque, or fibrous plaque. This study showed that the defined areas indeed had varying heteroplasmy level. Mutations G12315A and G14459A were strongly associated with primary atherosclerotic lesions, fibrous plaques, and total atherosclerotic lesions of the intimal segments. Mutation C5178A was strongly associated with fibrous plaques and total atherosclerotic lesions. Interestingly, mutations A1555G and G14846A negatively correlated with atherosclerotic lesions (at *p* ≤ 0.05 level of significance), in contrast to previous studies.

Therefore, the accumulating evidence demonstrates associations between several mtDNA mutations and atherosclerotic plaques at different stages of development. This confirms the important role that mitochondria are playing in atherosclerotic lesion development. Indeed, increased oxidative stress inside the plaques, which can be linked to mitochondrial dysfunction caused by mtDNA mutations is currently regarded as one of the components of atherosclerosis pathogenesis. However, the abovementioned studies were limited by the number of patients and the lack of functional analysis. It would be interesting to study how the mitochondrial function is affected by each of the identified mutations.

### 2.2. mtDNA Mutations Discovered in Studies Using Human Blood Samples

Data obtained from blood cells (peripheral blood lymphocytes) demonstrated the relationship of bother mtDNA contents and certain mtDNA mutations with cardiovascular diseases. Studies of blood cells could be performed on relatively large numbers of patients and healthy volunteers. A study conducted in China on 378 CAD patients and the same number of matched healthy controls demonstrated that patients had lower overall content of mtDNA in peripheral blood lymphocytes [16]. Therefore, low mtDNA was identified as a possible risk factor of CAD. A more recent study, also conducted in China, including 1511 CAD patients and 1553 matching control subjects, has confirmed that a low mtDNA copy number in circulating leukocytes was an independent risk factor of CAD [17]. Each 1-standard deviation decrease of mtDNA copy number was associated with a 1.14-fold increase of the CAD risk (*p* < 0.001) after adjusting for confounders. The authors mentioned oxidative stress as the possible reason of the observed relationship.

Point mtDNA mutations in blood cells associated with CVD are being actively studied, and the list of identified mutations is growing. Originally detected in atherosclerotic lesions of abdominal aorta, the “common deletion” mtDNA4977 was further investigated in leukocytes from patients with CAD and was found to be present in leukocytes with significantly higher incidence (five-fold) in CAD patients than in healthy individuals (26.2% vs 4.5% patients) [18]. Another study, performed on blood samples from type 2 diabetes patients and healthy subjects, showed the accumulation of somatic mutation A3243G in mtDNA with patient’s age and duration of diabetes. It was concluded that A3243G may serve as a marker of diabetes duration and atherosclerosis [19]. This conclusion was supported by a more recent study on type 2 diabetes mellitus patients with carotid atherosclerosis, in which the frequency of this mutation was higher in the carotid atherosclerosis group compared to the control group [20].

Study of correlation between the level of heteroplasmy for point mutation C3256T in human leukocytes and the presence of CAD was conducted using peripheral blood of 191 healthy individuals and patients with confirmed diagnosis [21]. Atherosclerotic lesions were detected and evaluated by means of high-resolution B-mode ultrasonography. The mutation was detected in all study participants, but the mean level of heteroplasmy differed significantly between healthy and patient groups, being 21.7% and 28.8%, respectively (difference was significant at *p* = 0.035). The mean levels of C3256T heteroplasmy were 16.8%, 23.8%, 25.2%, and 28.3% in individuals with low, moderate, significant, and high predisposition to atherosclerosis, respectively. In addition, heteroplasmy values closely correlated with individuals’ age (*r* = 0.296; *p* < 0.001).

The list of leukocytes mtDNA mutations associated with carotid atherosclerosis was enlarged by the results of another study that used human whole venous blood samples [22]. Apart from the abovementioned C3256T, heteroplasmy levels for several other single nucleotide changes (T3336C, G12315A, G13513A, G14459A, G14846A, and G15059A) were associated with the size of carotid atherosclerosis plaques assessed using quantitative ultrasound examination. Noteworthy, mutation A1555G did not correlate with the size of atherosclerotic plaques. The level of heteroplasmy correlated with patient’s age for mutations C3256T, C5178A, G12315A, G13513A, G14459A, and G15059A.

In another study, leukocytes were collected from 156 apparently healthy individuals who underwent the assessment of subclinical carotid atherosclerosis by high-performance ultrasonography and quantitative measurement of common carotid artery intima-media thickness [23]. The obtained results showed significantly higher heteroplasmy levels for C3256T, G12315A, and G15059A in patients with intima-media thickening and atherosclerotic plaques in the carotid artery as compared to individuals without these conditions. This was not the case for the mutations G13513A and Ins652G.

The impact of atherosclerosis is generally greater in older age groups. Therefore, the attempt to detect the correlation between mtDNA mutations and patient’s age was made in a study on leukocytes from 700 individuals [24]. The following point mutations were shown to have a strong positive correlation with age according to the heteroplasmy level analysis: G12315A, G14459A, G15059A (*p* ≤ 0.05). It was suggested that these mutations may contribute to the ageing process.

Another study focused on the peripheral blood leukocytes of a cohort of females with subclinical atherosclerosis [25]. Statistical analysis revealed five heteroplasmic mutations associated with pathological increase of intima-media thickness: G13513A, C3256T, G14709A, G14846A, and G12315A. Interestingly, G13513A, C3256T, and G14709A possibly contribute to development of atherosclerosis, whereas G14846A and G12315A, on the contrary, may have a protective effect. In addition, a high level of heteroplasmy for these nucleotide replacements was noted simultaneously and they have high coefficients of collinearity: Variance inflation factor (VIF) from 1.042 to 1.429. It was suggested that the mutations may be present on the same haplotypes of mitochondrial genome associated with atherosclerosis.

In another study conducted on whole blood samples from healthy subjects and patients with ultrasonographically detected atherosclerotic lesions in carotid arteries, a number of homoplasmic and heteroplasmic mutations in mtDNA associated with atherosclerosis were identified [26]. Homoplasmic mutations included G8251A, T204C, C12705T, and G3010A, while heteroplasmic were missense mutation G9477A and insertion 8528insA. They occurred more frequently in patients with atherosclerotic lesions compared to the control group. Noteworthy, the authors did not detect any previously known mtDNA replacements, which can be explained by the different new generation sequencing (NGS) platform used in this study (Roche 494 GS Junior Titanium system).

A comparative evaluation of mtDNA changes in blood samples from patients with CHD and history of myocardial infarction and healthy subjects was conducted using TaqMan RT-qPCR [27]. The authors concluded that the heteroplasmy levels of the following four mitochondrial mutations were associated with atherosclerosis: C3256T, G12315A, G13513A, and G15059A. Since the first two of them are located in genes coding for tRNA-Leu, and the last two are within the sequences of coding for NADH dehydrogenase subunit 5 and cytochrome B, respectively, they may cause defects in protein chains of respiratory enzymes and tRNA generated in the mitochondria leading to mitochondrial dysfunction. In another study, which was performed on blood samples from healthy persons and patients with asymptomatic atherosclerosis, seven homoplasmic mutations in mtDNA more frequently detected in atherosclerosis were identified: T204C, G228A, G1719A, G3010A, G8251A, C12705T, and C16223T [28]. The obtained data partially support the previous findings. A list of homo- and heteroplasmic mutations in mtDNA genes associated with atherosclerosis is provided in Table 1.

One interesting recent study was conducted in Poland on a group of 415 individuals that included healthy controls and patients with type 2 diabetes mellitus, obesity, or atherosclerosis [29]. The authors used both the traditional analysis of mtDNA mutations and a specially developed algorithm, MutPred, to assess the contribution of mtDNA variants to the pathology development. The authors observed a possible association between mtDNA variants that were mildly deleterious and atherosclerosis. Most interestingly, these mildly deleterious variants could have a synergetic effect on the disease development, so individuals harboring several different mtDNA mutations may have an increased risk of CVD development. In line with previously published studies, the authors suggested that the mtDNA variants may act through altering the electron transport chain functioning and increasing ROS production.

## 3. Conclusions

Altogether, the results of the studies described in this review prove the existence of a link between the changes in mtDNA and atherogenesis. As it could be expected, the mutations were identified in the genes encoding for the electron transport chain enzymes, mitochondrial ribosomes, and tRNA. However, the exact mechanisms of the involvement of these genes in atherogenesis at the cellular and molecular level remain unknown. It is suggested that these mutations may impair electron transfer and protein synthesis in the mitochondria, thus causing ROS production and subsequent oxidative stress, which is one of the key processes in atherogenesis. Due to high frequency of atherosclerosis in modern society, there is a need for reliable prognostic and diagnostic molecular makers to provide medical assistance to both individuals with high predisposition to this disease and patients already affected by atherosclerosis and associated diseases. Further mechanistic studies evaluating correlation between mtDNA changes in blood cells and atherosclerosis are needed.

## Figures and Tables

**Table 1:** Mitochondrial DNA (mtDNA) mutations associated with atherosclerosis.

Gene or Region	Mutation	Homoplasmic/ Heteroplasmic	MAF	Variant Allele fraction, Mean	Pro-atherogenic	Anti-atherogenic
D-loop	T204C	Homoplasmic non-coding	0.059	-	+	
	G228A	Heteroplasmic non-coding	0.059	-	+	
	C16223T	Homoplasmic non-coding	0.074	-	+	
MT-RNR1 (12S rRNA)	A1555G	Heteroplasmic	-	0.300		+
	ins652G	Heteroplasmic	-	0.105		+
MT-RNR2 (16S rRNA)	G1719A	Homoplasmic	0.059	-	+	
	G3010A	Homoplasmic	0.206	-	+	
MT-TL1 (tRNA-Leu (codon recognized UUR))	C3256T	Heteroplasmic	-	0.195	+	
	A3243G	Heteroplasmic	-	0.024	+	
MT-TL2 (tRNA-Leu (codon recognized CUN))	G12315A	Heteroplasmic	-	0.273	+	
MT-ND1	T3336C	Heteroplasmic synonymous	-	0.105	+	
MT-ND2	C5178A	Heteroplasmic nonsynonymous: Leu → Met	-	0.131	+	
MT-ND5	C12705T	Homoplasmic	0.059	-	+	
	G13513A	Heteroplasmic nonsynonymous: Asp → Asn	-	0.269	+	
MT-ND6	G14459A	Heteroplasmic nonsynonymous: Ala → Val	-	0.139	+	
MT-COX2 (Cytochrome C oxidase subunit 2)	G8251A	Homoplasmic	0.074	-	+	
MT-COX3 (Cytochrome C oxidase subunit 3)	G9477A	Heteroplasmic nonsynonymous: Val → Ile	-	0.023	+	
MT-ATP8 (ATP synthase subunit 8)	ins8528A	Heteroplasmic frame shift	-	0.062	+	
MT-TE (tRNA-Glu)	G14709A	Heteroplasmic	-	0.019	+	
MT-CYTB (Mitochondrially encoded cytochrome B)	G14846A	Heteroplasmic nonsynonymous: Gly → Ser	-	0.201		+
	G15059A	Heteroplasmic stop codon	-	0.299	+	

MAF, minor allele frequency.
